# ST Segment Elevation with Normal Coronaries

**DOI:** 10.1155/2016/3132654

**Published:** 2016-06-15

**Authors:** Pooja Sethi, Ghulam Murtaza, Ashwini Sharma, Timir Paul

**Affiliations:** ^1^Department of Cardiology, East Tennessee State University, Johnson City, TN 37604, USA; ^2^Department of Internal Medicine, East Tennessee State University, Johnson City, TN 37604, USA; ^3^Department of Internal Medicine, UAB, Montgomery, AL 36116, USA

## Abstract

Noncardiac causes should be kept in the differential while evaluating ST elevation on EKG. Rarely abdominal pathologies like acute pancreatitis can present with ST elevation in the inferior leads. Once acute coronary syndrome is ruled out by emergent cardiac catheterization alternative diagnosis should be sorted. Abdominal pathologies, like acute pancreatitis and acute cholecystitis, can present with ST elevation in the inferior leads. Treating the underlying condition would result in resolution of these EKG changes.

## 1. Introduction

Chest pain accounts for around 6 million annual visits to emergency department (ED) in the United States (US) [1]. Electrocardiogram and troponin level are one of the common modalities used to rule out any acute coronary events in these patients. Patients with abnormal electrocardiogram (EKG), troponin level, and risk factors are referred for undergoing coronary catheterization. In one study 26% of angiography done in acute phase of suspected STEMI was normal [2]. Chest pain in these cases might be secondary to other cardiac conditions other than acute coronary events or extracardiac causes. We present three such cases with extracardiac cause of abnormal EKG and troponin levels who had normal coronaries on catheterization.

## 2. Case  1

**Figure 1:**
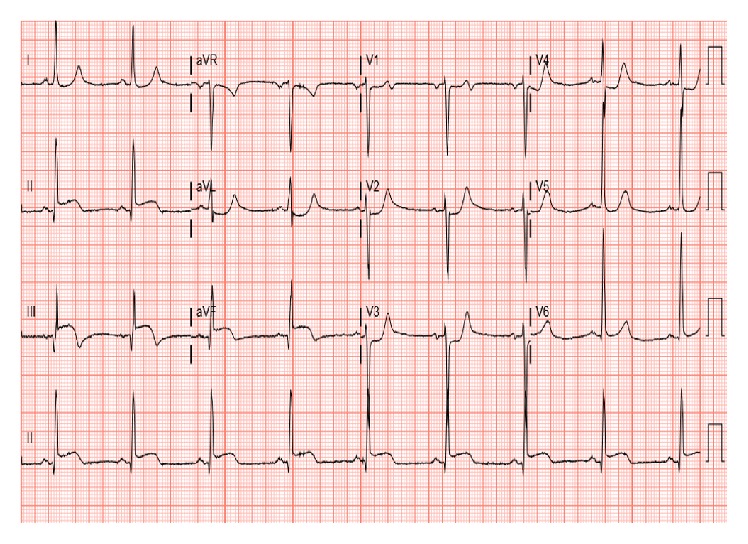
2 mm ST elevation in leads II, III, and AVF.

**Figure 2:**
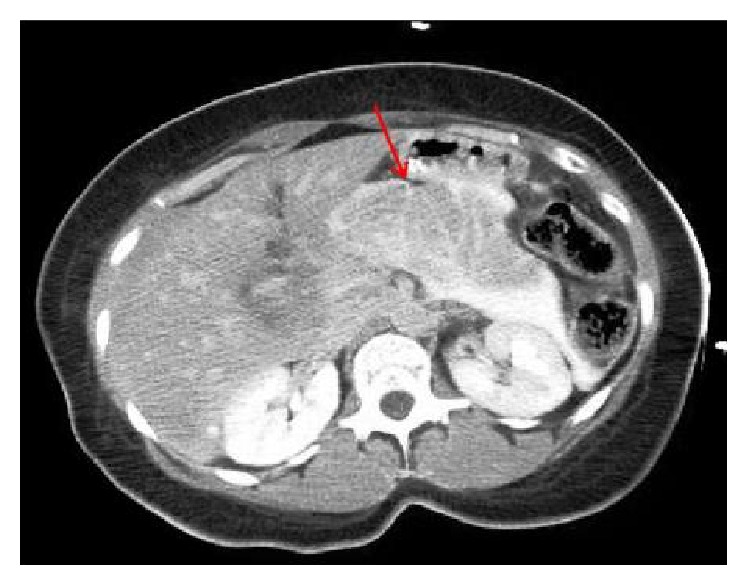
CT abdomen with contrast image showing swollen inflamed, edematous pancreas.

56-year-old lady with a history of hypertension, dyslipidemia, and gallstones presented to the emergency department (ED) with severe stinging, nonradiating, midsternal chest pain ongoing for 45 minutes. She experienced nausea, vomiting, and diaphoresis. She denied palpitations, orthopnea, or paroxysmal nocturnal dyspnea. On physical exam she was hypotensive with BP of 80/50 mmHg and a heart rate of 117 bpm. Her lungs were clear and cardiac exam showed normal heart sounds with no murmurs or rubs. She had mild epigastric tenderness, but there was no guarding or rigidity and bowel sounds were heard. 12-lead electrocardiogram (EKG) showed 2 mm ST elevation in leads II, III, and AVF, with reciprocal changes in leads I and AVL (Figure 1). With the diagnosis of acute inferior ST elevation myocardial infarction, STEMI protocol was initiated and emergent heart catheterization showed minimal coronary disease without the need for percutaneous intervention. Her labs data returned in the interim with WBC of 12,000 with no shift, hemoglobin of 11 g/dL, hematocrit of 36%, potassium of 2.6 mg/dL, magnesium of 1.4 mg/dL, and calcium of 7.2 mg/dL. Her serial troponins were 6.0, 1.2, and 0.87. Her liver function tests (LFT) were elevated alanine transaminase (ALT), 398 U/L; aspartate transaminase (AST), 863 U/L; alkaline phosphatase (ALP), 328 U/L; and lipase, 9,080 U/L. Computed tomography (CT) of the abdomen showed diffusely edematous and inflamed pancreas (Figure 2). She was started on intravenous fluids and pain medicine and received nothing by mouth to treat her acute pancreatitis. Interestingly, on repeat EKG, the inferior ST elevations had resolved. Over the course of her hospital stay her chest pain improved and her biochemical markers, including LFT and lipase, trended downwards, and she was discharged.

## 3. Case  2

**Figure 3:**
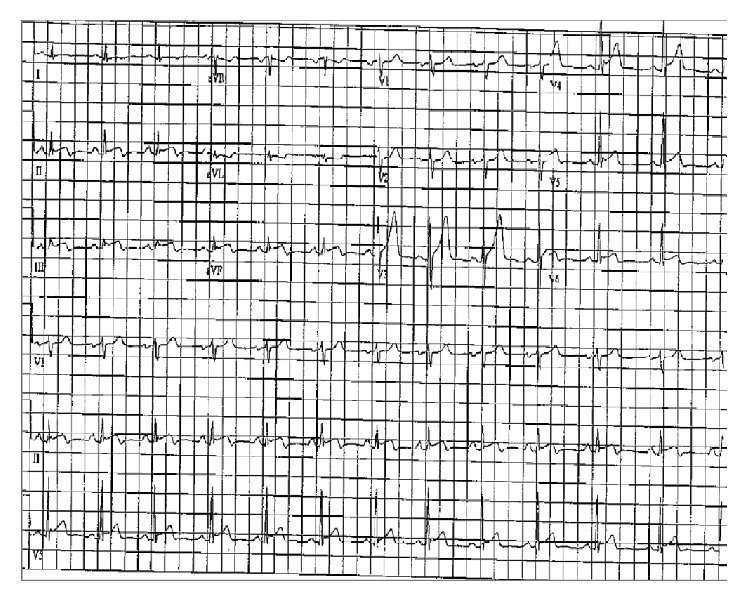
EKG showing ST elevation leads II, III, and AVF.

**Figure 4:**
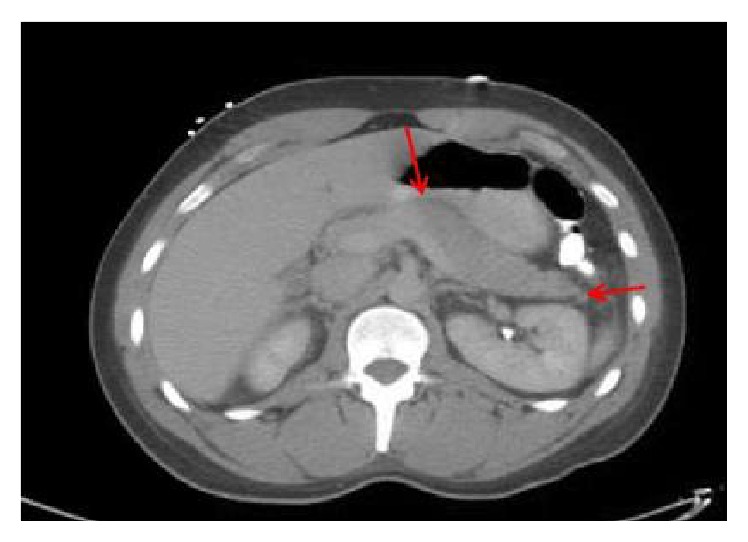
CT of the abdomen with contrast showing swollen pancreas and peripancreatic stranding.

28-year-old gentleman, with no significant past medical history, was transferred from outside hospital with diagnosis of STEMI. He had presented to the outside facility with severe, sharp, stabbing, midsternal chest pain and epigastric pain radiating to his axilla and back. His social history was significant for daily alcohol use with an average of 5-6 beers/day. He admitted to binge drinking one day prior to admission. EKG at the outside facility showed ST segment elevation in leads II, III, AVF, V5, and V6 (Figure 3). He had troponin elevation up to 4. In our hospital he was taken for emergent cardiac catheterization which showed normal coronaries. In the interim other tested lab work showed lipase of 4256 units/L. CT of the abdomen was significant for peripancreatic stranding consistent with the diagnosis of acute pancreatitis (Figure 4). He was treated with intravenous fluid hydration and pain medicines. His serial troponins trended down to 1.95 and 0.86. His repeat EKG did not show ST elevation. Patient was discharged in stable condition.

## 4. Case  3

**Figure 5:**
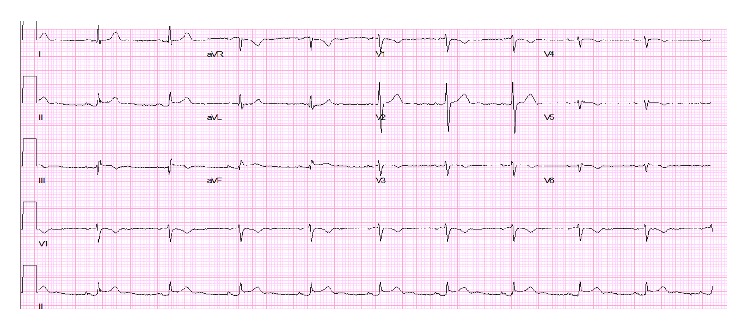
Showing ST elevation in II, III, and AVF.

**Figure 6:**
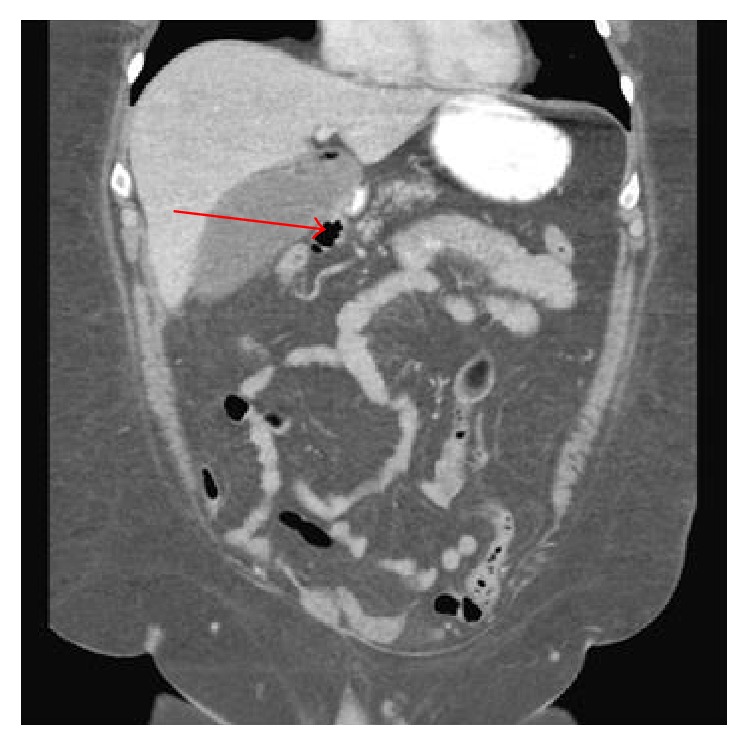
Arrow pointing to air in the gall bladder showing emphysematous cholecystitis.

A 43-year-old lady with history of hypertension, gastroesophageal reflux, and morbid obesity presented to the ED with severe knife-like chest pain and abdominal pain which started one hour prior to presentation. On physical exam her BP was 200/100 mmHg, HR was 96 bpm, RR was 22 breaths, and temperature was 100.5 F. She was alert but looked pale and diaphoretic. On cardiac exam there were no murmurs. Abdominal exam revealed positive murphy sign and right upper quadrant tenderness. EKG showed ST segment elevation in leads II, III, and AVF with reciprocal changes in the lateral leads (Figure 5). Her troponins were minimally elevated at 0.66. STEMI protocol was initiated and she was taken for urgent cardiac catheterization in keeping with the door to balloon time of 90 minutes. Cardiac catheterization revealed normal coronaries. Other lab work done showed WBC count of 14,000; liver enzymes, amylase, and lipase were within normal limits. CT scan of abdomen was done given right upper quadrant pain which revealed emphysematous cholecystitis with no gall stones (Figure 6). Surgery was consulted and she underwent a cholecystectomy. Repeat EKG no longer showed the ST elevation. The patient was discharged in stable condition.

## 5. Discussion

It is imperative to rule out cardiac ischemia in patients who present with chest pain and ST segment elevation on EKG. Emergent cardiac catheterization is the standard of care. However, once a cardiac etiology is ruled out other diagnosis should be sought.

A 2.3% incidence of alternative conditions mimicking STEMI is found in patients referred for primary percutaneous coronary intervention (PCI) [3]. Various multisystem etiologies include cerebrovascular conditions like subarachnoid hemorrhage, pulmonary conditions such as pneumonia, pulmonary embolism, and COPD, and nonischemic cardiac causes like myocarditis, myopericarditis, and acute heart failure (Table 1). Abdominal causes like acute pancreatitis, cholecystitis, and peritonitis comprise 5% of cases of noncardiac STEMI mimics [3, 4].

Abdominal etiologies like pancreatitis and cholecystitis can also cause troponin elevation. Release of pancreatic proteolytic enzymes causing transient myonecrosis and coronary vasospasm has been the hypothesized pathophysiologic mechanism [5]. Various other medical conditions can cause mild troponin elevation as well. These include nonischemic cardiac causes like myopericarditis, acute heart failure, defibrillator shocks, and arrhythmias. Pulmonary causes include pulmonary embolism. Systemic causes like septic and anaphylactic shock can also cause troponin elevation (Table 2) [6–9, 13–16]. This is usually secondary to left ventricular strain and demand ischemia rather than a coronary blockage.

The electrocardiographic changes in patients with acute cholecystitis and pancreatitis which have been reported are mostly transient [4, 10–12]. These changes usually are in the form of T-wave inversion, ST segment depression, and rarely ST segment elevation in the absence of coronary artery disease. The EKG changes in these patients are hypothesized to be secondary to changes in vagal nervous system. This also explains why EKG changes of ST elevation are mostly seen in inferior leads [10]. Other suggested mechanisms include cardiobiliary reflex, release of pancreatic proteolytic enzymes causing myonecrosis, transient coronary vasospasm, and any associated metabolic disturbances [4, 11, 12]. These EKG changes mostly resolve with the treatment of the underlying condition.

We did not perform echocardiogram in ED for our cases as emergent left heart catheterization was performed based on clinical presentation, EKG findings, and elevated troponins. However, echocardiogram can be helpful to improve the diagnostic specificity in selected cases. In patients having atypical chest pain, with ST elevation on EKG and/or troponin elevation where there is a clinical dilemma whether these patients need urgent catheterization or initial conservative management, echocardiographic assessment of ventricular function and wall motion abnormalities can help in further management. Absent wall motion abnormalities in patient with ongoing chest pain puts acute ST segment elevation myocardial infarction very low on differential. However, this must be balanced against the time delay necessary to obtain transthoracic echocardiogram and potential for ongoing myocardial injury.

## 6. Conclusion

Physicians should therefore consider intra-abdominal etiologies when faced with transient inferior ST segment elevations in patient without significant coronary disease.

## Figures and Tables

**Table 1:** Causes of ST segment elevation [1–5].

Neurological	Subarachnoid hemorrhage
Coronary	Coronary artery aneurysm, coronary artery occlusion, coronary artery stenosis, spontaneous coronary artery dissection, prinzmetal angina, cocaine abuse

Cardiac	Pericarditis, myocarditis, perimyocarditis, Brugada syndrome, left ventricular hypertrophy, left bundle branch block, cardioversion, takotsubo cardiomyopathy, cardiac compression

Vascular	Aortic dissection, pulmonary thromboembolism

Pulmonary	Pneumonia, COPD, mediastinal tumor

Abdominal	Cholecystitis, pancreatitis, hiatal hernia, subdiaphragmatic abscess, peritonitis

Electrolytes	Hyperkalemia, hyper/hypophosphatemia

Endocrine	Pheochromocytoma, thyroid storm

**Table 2:** Causes of elevation of troponins [6–12].

Nonischemic cardiac	Myocarditis, congestive heart failure, cardiac amyloidosis, cardiac contusion, closure of atrial septal defect, cardioversion and defibrillator shocks, supraventricular tachycardia

Pulmonary	Pulmonary thromboembolism

Abdominal	Cholecystitis, pancreatitis

Renal	Chronic renal failure

Neurological	Subarachnoid hemorrhage, stroke

Systemic	Septic shock, sepsis, critically ill patient, scorpion envenoming, high dose chemotherapy
